# Exploring the Role of Enhancer-Mediated Transcriptional Regulation in Precision Biology

**DOI:** 10.3390/ijms241310843

**Published:** 2023-06-29

**Authors:** Xueyan Wang, Danli Liu, Jing Luo, Dashuai Kong, Yubo Zhang

**Affiliations:** 1 College of Life Science and Technology, Huazhong Agricultural University, Wuhan 430070, China; wangxueyan@caas.cn; 2 Shenzhen Branch, Guangdong Laboratory of Lingnan Modern Agriculture, Key Laboratory of Livestock and Poultry Multi-Omics of MARA, Agricultural Genomics Institute at Shenzhen, Chinese Academy of Agricultural Sciences, Shenzhen 518120, China; liudanli@caas.cn (D.L.); luojing02@caas.cn (J.L.); kongdashuai@caas.cn (D.K.)

**Keywords:** enhancer-mediated, transcriptional regulation, precision biology, three-dimensional (3D) genomics

## Abstract

The emergence of precision biology has been driven by the development of advanced technologies and techniques in high-resolution biological research systems. Enhancer-mediated transcriptional regulation, a complex network of gene expression and regulation in eukaryotes, has attracted significant attention as a promising avenue for investigating the underlying mechanisms of biological processes and diseases. To address biological problems with precision, large amounts of data, functional information, and research on the mechanisms of action of biological molecules is required to address biological problems with precision. Enhancers, including typical enhancers and super enhancers, play a crucial role in gene expression and regulation within this network. The identification and targeting of disease-associated enhancers hold the potential to advance precision medicine. In this review, we present the concepts, progress, importance, and challenges in precision biology, transcription regulation, and enhancers. Furthermore, we propose a model of transcriptional regulation for multi-enhancers and provide examples of their mechanisms in mammalian cells, thereby enhancing our understanding of how enhancers achieve precise regulation of gene expression in life processes. Precision biology holds promise in providing new tools and platforms for discovering insights into gene expression and disease occurrence, ultimately benefiting individuals and society as a whole.

## 1. Introduction

For many years, scientists have faced challenges in solving biological problems with precision and efficiency. Therefore, precision biology has emerged as a field that provides more efficient and accurate means for life science research [1,2]. Precision biology is a discipline focused on elucidating the biological effects of genes, proteins, and other biomolecules through the analysis of their intricate interactions. It has evolved from the field of precision medicine and extends beyond the realm of human biology. Precision biology investigates the molecular pathways that underlie disease onset and progression by considering individual variations in genes, environment, and lifestyle factors [3,4,5]. It can help researchers to identify specific targets for the development of drugs and the design of personalized treatment regimens [6,7,8]. Complementing precision biology, recent advances in three-dimensional (3D) genomics enable researchers to reveal the overall pattern of transcriptional regulation of genes on a genome-wide scale [9,10]. This clarification helps identify the regulatory elements required to transcribe specific genes and understand how they interact with other genes [9,10].

Transcriptional regulation, as a determinant in gene expression, is accompanied by a large number of changes in regulatory elements and is particularly important in precision biology [11]. Genes exhibit different transcriptional patterns, such as expression or shutdown, when interacting with different transcriptional elements or protein molecules. The gene expression level also increases or decreases continuously with changes in the concentration of transcriptional elements or protein molecules in the gene expression pattern [12]. 3D genomics reveals the interactions between different genes and transcriptional elements in cells and even organisms, which can be used to determine the specific spatiotemporal expression patterns of genes that may depend on these interactions [12]. These minor changes in the expression pattern of intranuclear target genes caused by different interactions of genes or transcription elements can be directly used in the study of precision biology and will directly advance the development of precision biology. In this context, special attention is given to enhancers, which are regulatory elements involved in enhancer-mediated transcriptional regulation.

There is a large number of non-coding DNA and RNA regulatory elements in the genome, and enhancers are one type of non-coding DNA sequences [13,14]. They are mainly found in intergenic and intronic regions and activate the expression of target genes [15,16]. As cis-regulatory DNA elements in eukaryotes, enhancers bind tissue-specific transcription factors (TFs) and specifically transmit regulatory information to their target gene promoters, participating in transcriptional regulation [17,18]. Through continuous research and accumulated datasets, scientists have realized that the number of enhancers is far greater than the number of genes [19,20,21]. Enhancer-mediated transcriptional regulation is tightly correlated with spatiotemporal gene expression patterns. Enhancers are crucial for the proper development and function of organisms, as they determine the specificity of cellular responses to environmental stimuli and regulate biological processes at the molecular level [22]. However, there is still a lack of studies on the regulation of the same gene by multiple enhancers at different stages of growth and development. It is essential to resolve the patterns of multi-enhancer regulation. In recent years, super enhancers have also received more attention due to their key role in controlling cellular identity and disease, especially cancer [23,24,25]. Super enhancers are clusters of enhancers that have more complex mechanisms of action, providing a more appropriate explanation for fine transcriptional regulation. In other words, researchers can start with enhancers to explore the three-dimensional spatial conformational information and study the different functions of genes. This approach can help dissect the possible patterns of spatio-temporal specificity in gene expression within transcriptional regulator networks.

Herein, we summarize the research progress, focusing on the importance of enhancer-mediated transcriptional regulation in achieving precision biology. Specifically, we highlight the multi-enhancer transcriptional regulation model, which offers a new approach to studying the function and molecular mechanism of individual genes [26]. We hope to provide novel insights into the study of multi-enhancer regulatory models, enhancing our understanding of the mechanisms involved in integrating information through complex devices in transcriptional regulation. By doing so, we aim to advance the field of precision biology. Moreover, it also has a positive impact on our future understanding of diseases and the development of personalized medicine.

## 2. The Development of Precision Biology

Precision biology has emerged from precision medicine, aiming to gain a more complete and accurate understanding of biological systems through the utilization of advanced technologies and methods. The concept of precision medicine was introduced by the National Institutes of Health (NIH) in 2015, with the goal of providing personalized information to individuals and their families to improve health outcomes [27]. Precision medicine represents a novel approach to disease prevention and management [1,28] that considers individual genetic, environmental, and lifestyle differences. Its primary focus is on treating cancer, while the long-term objective is to establish a new model of medicine that emphasizes individualized engagement, data sharing, and privacy protection [29,30]. The integration of artificial intelligence (AI) and machine learning (ML) makes it possible to employ computational approaches in precision medicine [31,32]. These approaches enable the identification of drugs that can be repurposed for other diseases, utilizing diverse sources of data ranging from molecular signatures to the effects of drugs at the molecular and system levels. In precision biology, advanced technologies and methods are employed, including high-throughput experimental techniques, such as genome sequencing, proteomics, metabolomics, and single-cell analysis. These techniques generate large amounts of data, which can then be analyzed using computational methods [27,33].

**Figure 1:**
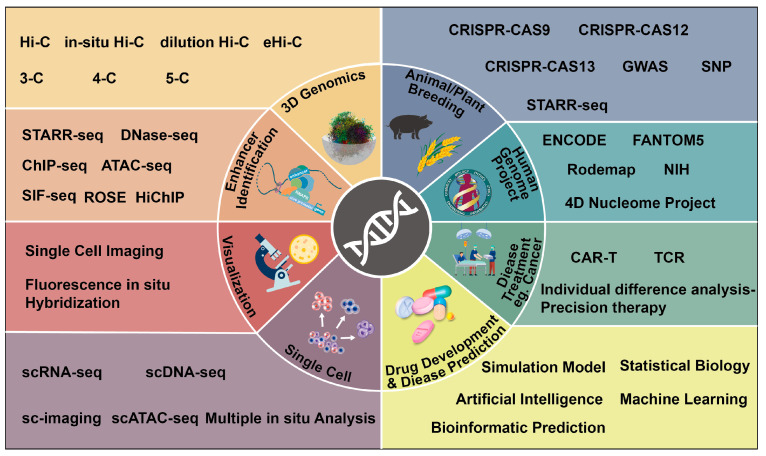
List of technologies that promote the development of precision biology. The multidimensional resolution of precision biology (inner circle), the advancement of precision biology through various enabling milestones through different translational axes, such as high-throughput research methods and big data analysis and integration (outer frame).

In the previous section, we mentioned that subtle changes in gene expression patterns resulting from interactions between genes and transcriptional elements (especially enhancers) play a direct role in precision biology research. To investigate these “minor changes”, a number of 3D genomics techniques have been developed. These include chromosome conformation capture (3C)-based technologies, such as chromosome conformation capture-on-chip (4C) [34], chromosome conformation capture carbon copy (5C) [35], high-throughput/resolution chromosome conformation capture (Hi-C) [36], Single-cell Hi-C [37], eHi-C [38], in 3D genomics, chromatin interaction analysis by paired-end tag sequencing (ChIA-PET) [39], and related methods, which have enabled the discovery of transcriptional regulatory elements, especially enhancers, and their effects on gene expression (Figure 1). In addition, various methods, such as self-transcribing active regulatory region sequencing (STARR-seq), chromatin immunoprecipitation sequencing (ChIP-seq) [40,41], DNase-seq, site-specific integration FACS-sequencing (SIF-seq), assay for transposase accessible chromatin with high-throughput sequencing (ATAC-seq), robust statistical estimation (ROSE) algorithm, in situ Hi-C followed by chromatin immunoprecipitation (HiChIP), have been widely used for the identification and annotation of enhancers and super enhancers (annotations of enhancer and super enhancer are described in detail in Section 4). These techniques significantly contribute to the development of 3D genomics.

For instance, researchers have utilized Hi-C and ChIP-seq to investigate the impact of MED23 mutations on enhancer activation and chromatin conformation. These studies have provided insights into the pathogenic mutations of MED23 and the role of mediator in transcriptional control based on enhancer topology [42]. Single-cell Hi-C has been used to systematically map the three-dimensional genomic and dynamic epigenetic profiles of macrophages during differentiation and immune response [43]. This approach has revealed the potential mechanisms underlying the reprogramming of gene expression profiles in immune cells (Figure 1). The SIF-seq has been developed to evaluate enhancer activity in various disease-associated cell types. It has successfully identified cardiac enhancers in in vitro differentiated cardiomyocytes and neuronal enhancers in neural progenitor cells [44], contributing to our understanding of disease-related mechanisms. STARR-seq has been instrumental in identifying regulatory variants associated with cancer susceptibility [45]. It has contributed to the interpretation of genome-wide association studies (GWAS) results and provided valuable information for cancer risk assessment. Moreover, these cutting-edge technologies have also been used to uncover potential target enhancers for agronomic traits in plants, such as salt stress tolerance [46], facilitating the achievement of precision crop breeding. These advanced techniques have significantly expanded scientists’ knowledge of the relationship between genome-wide spatial conformation and gene transcriptional regulation, thereby driving the progress of precision biology.

On the other hand, recent changes in sequencing technology have led to the rapid development of single-cell sequencing [37], enabling researchers to gain insights into cellular information at the individual cell level and creating new opportunities for studying enhancer-mediated transcriptional regulation. Single-cell omics and imaging techniques have become essential tools for the realization of precision biology [47,48,49]. These techniques, including single-cell RNA sequencing (scRNA-Seq), single-cell DNA sequencing (scDNA-Seq), and single-cell assay for transposase accessible chromatin with high-throughput sequencing (scATAC-Seq), as well as various in situ analysis methods, allow for the measurement of gene expression, mutations, epigenetics, and open chromatin regions. Such information is crucial for resolving tumor heterogeneity, characterizing the tumor microenvironment, and identifying rare cell subpopulations [50,51]. Furthermore, advancements in imaging techniques have facilitated the study of single-cell imaging, overcoming the limitations of single-cell histology techniques in capturing and visualizing the spatial relationships of cells within their native tissue environment [52]. Spatially single-cell epigenomics approaches have enabled the labeling of histone modifications of active promoters, putative enhancers, and silent promoters, enabling spatial analysis of epigenetic modifications and DNA-binding proteins [53]. These techniques have advanced our understanding of how gene expression is regulated spatially and temporally by the epigenome. By simultaneously capturing genomic output at different levels, single-cell techniques allow scientists to document cellular properties and functions [54,55]. Epigenomic technologies play a crucial role in understanding cellular diversity and uncovering mechanisms of gene regulation. This, in turn, contributes to the discovery of enhancer-mediated transcriptional regulation. It has been observed that there is a synergistic effect of tumorigenesis among super enhancers [56]. Therefore, understanding the mechanism of enhancer-mediated transcriptional regulation from a single-cell perspective can provide a new level of understanding of cell fate determination, identity, and function in normal development, physiology, and disease. Careful and comprehensive analysis of individual cells within a population will likely completely change the way we describe and treat disease and may even explain some of the different responses that occur in patients with similar conditions and treatment processes.

Developments in bioinformatics and computational biology have further improved data availability and accuracy, contributing to accurate screening enhancers and enhancer-promoter interactions from large sequencing datasets [57,58]. Furthermore, the Human Genome Project (HGP) [59], the Human Genome Encyclopedia (ENCODE) Project [13], and the ongoing 4D Nucleosome Project [60] are progressing well thanks to the infrastructure of systems biology (Figure 1). These initiatives provide valuable resources for precisely targeting, screening, and comprehensively characterizing the enhancer-mediated transcriptional regulation network.

The advancements in precision biology have yielded remarkable progress in medical research, leading to improved healthcare standards and overall public health. In the pursuit of precision biology, to explore the individual differences and mutations that exist in individual genomes and how these differences and mutations affect the physiological and pathological characteristics of cells, tissues, and individuals, transcriptional regulation, especially enhancer-mediated transcriptional regulation, serves as a crucial tool and platform for precision biology.

## 3. Transcriptional Regulation and Precision Biology

Transcriptional regulation is commonly believed that occurs at two interrelated levels: The first involves transcription factors and the transcriptional apparatus, while the second involves chromatin and its regulators, including enhancers. Researchers are currently striving to elucidate how mammalian gene expression is temporally and spatially constrained by integrating these two aspects [16,61]. Given the potential mediating role of enhancers between these two levels, we once again emphasize the importance of minor changes in gene expression patterns resulting from enhancer-mediated transcriptional regulation in precision biology studies.

### 3.1. Transcription Factors and Transcriptional Apparatus

In the first level, transcription factors and transcription apparatus, since the eukaryotic RNA polymerase itself has no specific affinity for promoters, numerous transcription factors and co-transcription factors are required to form a complex transcription apparatus [62,63,64]. In eukaryotes, precise regulation of gene expression depends on two critical components: An organization-specific transcription factor, which acts as the “commander” of a specific gene expression network, and a set of general-purpose machines, commonly known as “transcriptional machinery” that carry out the gene transcription process specified by the “commander” and produce the corresponding transcription products (mRNA). To achieve cellular and tissue specificity of gene expression, different transcription factors are used by the same transcription machinery. The mediator complex plays a central role in facilitating communication between the transcription factors and the transcription machinery [65,66,67,68]. Its role in transcriptional regulation has been refined and deepened by advances in high-throughput technologies and structural biology. The prevailing view in the field is that mediator plays a critical role in the transcriptional initiation and normal activation of almost all protein-coding genes. In transcription initiation, the core promoter not only facilitates the transcriptional initiation of transcription by the basal transcription machinery but also functions as a regulatory element, and it would be interesting to determine the mechanistic basis of enhancer-core promoter specificity. Thus, resolving the composition and assembly of the transcriptional apparatus helps one to explain the spatiotemporal specificity of gene expression.

**Figure 2:**
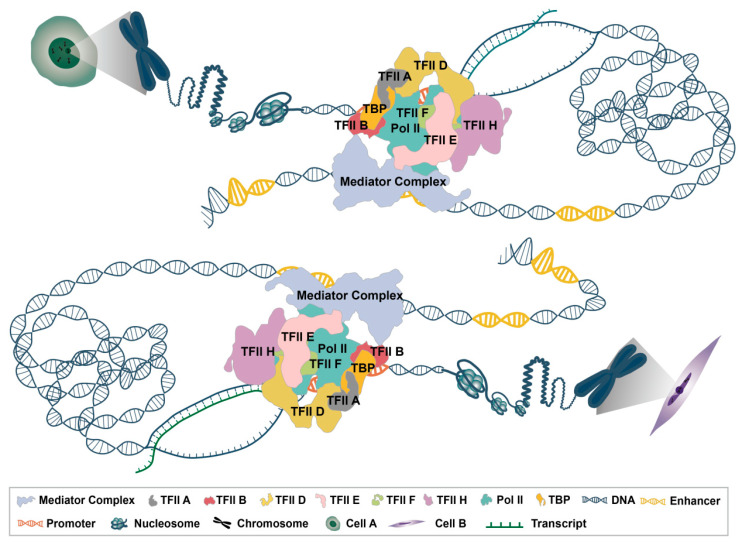
Transcription initiation complex binding enhancer and promoter. The enhancer (yellow sequence) binds to mediator complex of transcription initiation complex. It can interact with the promoter (orange sequence) bound to Pol II. The same promoter may bind different enhancers in different cells. The promoter interacts with the second enhancer in cell A and the same promoter interacts with the first enhancer in cell B.

RNA polymerase binds to the promoter to initiate transcription. Enhancer-promoter binding relies on a complex structure of cell type-specific TFs, coregulators, chromatin modifiers, architectural proteins like cohesin, condensin, and CCCTC binding factor (CTCF), other enzymes, and RNA polymerase II (RNAPII). The team of Anders S. Hansen utilized super-resolution live cell imaging to observe chromatin looping within the topologically associating domain (TAD) of *Fbn2* in mouse embryonic stem cells, revealing a highly dynamic CTCF/cohesin-mediated looping state that illustrates the presence of the TAD most often in a partially extruded state of cohesion [69]. Frequent cohesin or CTCF-CTCF-mediated contacts within the TAD (enhancer-promoter physical contacts) play a key role in the regulation of gene expression [69]. Preinitiation Complex (PIC) and enhancer-promoter linkage are also achieved through mediator’s large protein complexes. Recent studies have reported the structure of the TBP (TATA-binding protein) -based PIC-binding mediator complex [70,71], revealing the interaction between the two complexes. Mediator and TFII D, two core regulators, bind to the enhancer and promoter regions, respectively (Figure 2). However, it remains unclear how the two core regulators work together to promote PIC-mediator assembly at the promoter, regulate the phosphorylation of C-terminal domain of the large subunit of RNA polymerase II (Pol II CTD), and activate transcription. The complete TFII D-based PIC and mediator form a PIC-mediator complex containing all factors necessary for transcription initiation and complete basal transcription. Almost all transcription factors and regulators act on the PIC-mediator complex to activate transcription.

In April 2021, Yanhui Xu et al. solved the complete human mediator-bound preinitiation complex for the first time, demonstrating the dynamic assembly of the PIC-mediator and proposing a molecular model for how mediator regulates the phosphorylation of the Pol II CTD [72] (Figure 2). This breakthrough work addressed the central question of how mediator, a transcriptional co-activator, coordinates the assembly and activation of TFII D-based PICs. The study revealed that TFII D recognizes not only the TATA box through TBP but also downstream promoter elements through TBP-associated factors (TAFs), enabling the regulation of almost all protein-coding genes. This overturns the conventional view that TBP only binds to the TATA box at the molecular level and explains how PIC assembly and gene transcription can occur at the promoters of almost all genes. The proposed TFII D-based PIC-mediator structure is currently the most complete and accurate representation of the transcription initiation complex under physiological conditions. Core promoter elements contribute to the gene selectivity of enhancers, and the gene-specific activation of enhancers involves their communication with the underlying RNAPII transcriptional machinery of the core promoter [73]. Mediators can form a bridge by interacting with transcription factors on PICs and enhancers, thereby transmitting enhancer-to-promoter signals. Therefore, the proposed structure provides a theoretical foundation for understanding the mechanism of enhancer-core promoter specificity.

### 3.2. Transcriptional Regulatory Elements

The second level of transcriptional regulation involves chromatin and its regulators. Within complex genomes, transcriptional regulatory elements play a critical role in governing gene expression throughout development, maintaining cellular and tissue homeostasis, responding to external stimuli, and contributing to disease [74]. Although these elements do not encode proteins themselves, they serve as binding sites for trans-acting factors that ultimately control gene expression [74]. Numerous studies have investigated the functions of these cis-acting elements in transcriptional regulation.

The promoter is the most fundamental cis-acting element for transcriptional regulation and serves as the site of enhancer-mediated transcriptional regulation. The length and sequence of the promoter can vary widely depending on the gene, the type or class of RNA polymerase recruited, the transcription product type, and the species of the organism [75,76]. In vitro studies focusing on the RNA polymerase-promoter complex can be traced back to the 1980s [77,78]. TFs and RNAPII are recruited to the promoter sequence to guide the direction and precise initiation of transcription, while transcriptional enhancers activate or enhance the expression of their target genes [79,80,81].

Although the precise molecular mechanism of enhancer-promoter (E-P) interaction remains poorly understood, it is evident that changes in E-P interactions can modulate the expression of target genes [82]. For example, in human leukemia cells, an increase in E-P contacts and the number of interacting regions within target gene promoters can influence the expression of oncogene [83]. Moreover, different E-P interactions involving the *Foxp3* transcription factor play a role in coordinating the development, maintenance, and function of regulatory T cells (Treg) [84]. However, several reports suggest that physical contact between enhancers and promoters alone is insufficient for transcription and does not solely rely on high levels of related transcriptional co-activators such as bromodomain containing 4 (BRD4) or mediator [85]. Nevertheless, dysregulation of BRD4, which corresponds to E-P interactions, can contribute to diseases, such as cancer [82]. Additionally, the formation of long-range E-P interactions depends on CTCF-induced DNA loops, which facilitate the recruitment of enhancers to specific promoters [86,87].

Scientists have made efforts to accurately characterize enhancer-mediated transcriptional regulation in order to determine the precision and accuracy of transcriptional location, level, and timing [10,88]. The three-dimensional folding of the mammalian genome plays a crucial role in controlling gene expression and cell fate during development and tumorigenesis. Interactions between gene-regulatory elements are often organized within insulated neighborhoods, and regulatory signals that induce transcriptional changes can reshape chromatin folding patterns and alter gene positioning within the nucleus [86,87]. This highlights the interdependence between transcriptional regulation and genomic organization. Therefore, understanding the mechanism by which enhancers precisely interact with their target promoters and avoid off-target effects is of utmost importance in the study of gene regulatory networks. This raises the question of how the mediator complex transmits the activation of gene expression by transcription factors.

### 3.3. Regulation of Chromatin Dynamics in Transcription

Upon activation of PIC-Mediator, transcription factors can recognize and bind upstream promoter elements and enhancers to exert regulatory effects on gene transcription (Figure 2). In this process, DNA needs to unwind from the core histones to allow transcription factors and transcription machinery to access DNA with RNA polymerase, a process known as chromatin remodeling [89]. This dynamic regulation of chromatin is of great importance for biological inheritance. Recently, Prof. Zhucheng Chen’s team reported the structure of the human-derived chromatin remodeling complex PBAF (polybromo-associated BRG1-associated factor) bound to nucleosomes in the active state [74], revealing the assembly of the PBAF complex consisting of 12 subunits and elucidated the mechanism by which the PBAF complex recognizes nucleosomes, providing a theoretical framework for understanding the pathogenesis of human disease-associated mutations. The regulation of histone degradation and abundance during chromatin remodeling is still an area of great interest. Changes in histones and histone modifications can also impact the three-dimensional structure of DNA, leading to interactions between transcriptional regulatory elements [90]. Additionally, last year, Arul M. Chinnaiyan’s team reported a protein hydrolysis-targeted chimeric degradation agent called AU-15330, which specifically targets the SWI/SNF (switch/sucrose non-fermentable) chromatin remodeling complex ATPases subunits SMARCA2 and SMARCA4 [91]. AU-15330 efficiently inhibits androgen receptor-dependent prostate cancer growth by inhibiting chromatin opening in the enhancer region mediated by the SWI/SNF chromatin remodeling complex. This finding suggests that targeting enhancer accessibility mediated by the SWI/SNF chromatin remodeling complex could be a promising therapeutic approach for enhancer-addicted cancers. Therefore, understanding the mechanism of enhancer-mediated transcriptional regulation will provide new insights into the regulation of chromatin dynamics.

## 4. The Role of Different Enhancers in Transcriptional Regulation

Compared to the intricate pathways and diverse transcriptional machinery involved in parsing transcriptional regulation, analyzing the differences in gene expression attributed solely to enhancers can provide insights into the role of transcriptional regulators from a three-dimensional genomics perspective. This approach can help identify transcriptional regulators to some extent.

### 4.1. Typical Enhancers

Typical enhancers (TEs) are short genomic DNA sequences. They are usually considered to have the following characteristics, including: Enhancing the expression of the target gene, the function independent of position, being located in the open chromatin region, exhibiting enrichment of transcriptional co-activators with histone modifications, and containing specific DNA sequences that allow for binding TFs [17]. The first enhancer discovered was a 72 bp-long DNA fragment from the late gene region of simian virus SV40, which increased the expression of a reporter gene promoter by ~200-fold [92,93].

### 4.2. Annotation of Typical Enhancers

To explain enhancer-mediated transcriptional regulation and understand E-P interactions, scientists need to systematically annotate enhancers. In the genomic era, various methods have been employed for enhancer annotation. For example, histone-modified ChIP combined with microarrays (ChIP-ChIP) and next-generation sequencing (ChIP-seq) to predict enhancers [94,95,96]. Currently, commonly used methods for enhancer annotation include: Integration of DNase hypersensitivity analysis with deep sequencing, detecting of a higher proportion of histone H3 lysine 4 monomethylation (H3K4me1) compared to trimethylation (H3K4me3), assessing histone acetylation (e.g., H3 acetylated at lysine 27 (H3K27ac)), identifying certain histone variants (e.g., H2AZ), examining the binding of co activator to acetyltransferases (e.g., creb-binding protein with p300 (CBP/p300)), and analyzing the aggregation binding of multiple TFs [79,96,97,98]. Thousands of enhancers in various animal models, including drosophila, nematodes, mice, and humans have been annotated by different international genome annotation consortia such as ENCODE [13,99], NIH Epigenome Roadmap [100], FANTOM5 [101,102], and Blueprint/IHEC [103]. Additionally, enhancer-related databases such as VISTA enhancer Browser [104], enhancer Atlas [105], and Human Enhancer Disease Database (HEDD). [106] have been developed to visualize and share enhancer annotation information among mammals (Figure 1). These resources provide valuable insights into the role and mechanisms of enhancer-mediated gene regulation. The use of epigenomic markers has shifted towards identifying developmental enhancers that are more likely to drive tissue-specific patterns of gene expression [95,107]. However, the annotation of epigenomic features has generated a large number of putative enhancers in humans (ranging from 400,000 to 1 million), which is more than ten times the number of coding genes. Importantly, the presence of histone modifications does not fully explain the molecular mechanisms underlying enhancer activity [95,108]. Arbitrary truncation based on the H3K4me1/H3K4me3 ratio to select enhancers may miss some functional enhancers [109]. These findings suggest the need for additional criteria to annotate functional enhancers more precisely in the genome.

### 4.3. Super Enhancer

**Figure 3:**
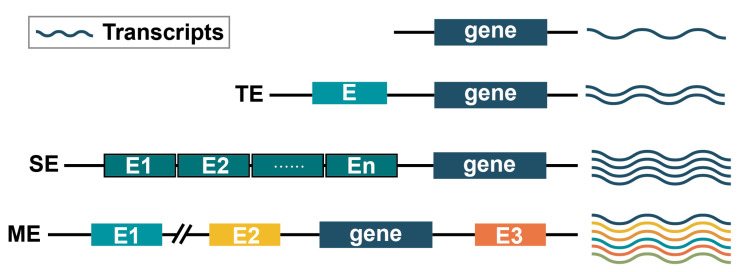
Comparison of typical enhancer (TE), super enhancer (SE) and multi-enhancer (ME) interoperability patterns. (1) TE usually positively drives target gene expression and more transcripts are generated. E denotes a single enhancer. (2) SE is usually a cluster of enhancers consisting of neighboring enhancers. SE has a stronger effect on gene transcription enhancement than TE. It can help to produce more transcripts. E1 to En denote different enhancers. (3) In ME modulation, enhancers may be distributed at different positions of the gene and may be far from the gene, such as E1 or be adjacent to the gene, such as E2. Each enhancer can enhance gene expression, but the resulting transcriptions may be different. Different transcriptions are shown in different colours.

Cells rely on 3D genomic organization for transcriptional control, and they also utilize super enhancers (SEs) to regulate transcription. SEs are a class of cis-regulatory elements with super-transcriptional activation properties. In 2013, Richard A. Young’s lab proposed the concept of SEs [23] based on the study of enhancers at that time. Super enhancer regions typically span 8–20 Kb, which is much larger than the 200–300 bp span of normal enhancers. They are a large cluster of transcriptionally active enhancers enriched with a high density of master transcription factors (Figure 3), cofactor, and enhancer histone modification marks that control cell identity gene expression [110,111]. These SEs can explain cell type-specific expression patterns [112] and have significant potential for applications in developmental biology [113], cancer, and other disease [113,114,115] pathogenesis studies.

#### 4.3.1. Comparison of Typical Enhancers and Super Enhancers

SEs play a similar role to Tes in mediating transcriptional regulation. Super enhancers differ from typical enhancers in terms of size, transcription factor density and content, and their ability to activate transcription [23] (Figure 3). It is generally believed that Ses have a higher density of H3K27ac and H3K4me1 modifications [23,111], and they bind the mediator complex and bromodomain containing four proteins [116]. Their binding of transcription factors and the marking of chromosomes associated with transcriptional activity are also much higher in SEs compared to TEs [110]. As a result, genes regulated by SEs are expressed at higher levels compared to those regulated by TEs, and their activity is more sensitive transcription factors blockade. Moreover, the individual enhancers that make up the SEs can activate gene transcription similar to TEs. SEs produce higher levels of eRNA than TEs [23,117]. For example, in toll-like receptor 4 (TLR4) signaling in macrophages, about 93% of the SEs and about 30% of the intergenic TEs are associated with eRNA [118]. Super enhancers and their associated genes are often located within loops co-occupied by two CTCF sites within TADs. As an example, 84% of SEs and 48% of TEs are located within such structures in mESCs [119].

#### 4.3.2. Annotation of Super Enhancers

Now ChIP-seq is the most commonly used technique for analyzing super enhancers. This method takes advantage of the enrichment of transcriptionally active markers, such as H3K27ac and H3K4me1, chromatin modification P300, near SEs. Antibodies specific to these active marker molecules are used in ChIP-seq to identify their enrichment patterns in the genome, thereby identifying potential active enhancer sites. The threshold value for SE identification is determined through enhancer stitching and sorting, and the ROSE algorithm is employed to identify super enhancers [23,120]. Additionally, other technologies can be combined with ChIP-seq to improve the detection rate of super enhancers. For instance, ATAC-seq [121]. In situ Hi-C followed by chromatin immunoprecipitation (HiChIP) [122] can also be employed to analyze the 3D structures of SEs and their target genes. Once SEs are identified, it becomes possible to predict the expression of protein-coding genes and non-coding RNAs regulated by these super enhancers based on their genomic locations. RNA-seg can be integrated with this information to correlate SEs with abnormally highly expressed mRNAs, lncRNAs, circRNAs, and miRNAs in diseases. This analysis can help infer key SEs that potentially target disease-related genes and guide further functional studies.

#### 4.3.3. Regulatory Network of Super Enhancers

SEs exhibit diverse target genes within the three-dimensional genome. The functionality and action mechanism of the same SE may vary among different tumor cells. For instance, a recent study identified a novel SE, known as EphA2-super-enhancer (EphA2-SE), which is present in various tumors [123]. EphA2-SE exerts an oncogenic role by recruiting different transcription factors to drive the expression of the oncogene EphA2, thereby promoting tumor progression. Another study, which applied integrated epigenomic and transcriptomic profiling, revealed heterogeneity in SEs. This heterogeneity provided clinically relevant biological insights specifically for triple-negative breast cancer (TNBC) [124]. Understanding the mechanisms by which SEs and their transcriptional components promote oncogenic transcription and cancer development is crucial in precision medicine. This knowledge can facilitate the development of targeted drugs that selectively act on oncogenic SEs, specific chromatin interactions, or cancer-associated condensates.

Recent studies have proposed a model for the regulation of gene expression by SEs through phase separation [116]. This study demonstrates that the transcriptional co-activators BRD4 and MED1 can bind at super-enhancers and regulate key gene expression by segregating transcriptionally relevant components from the complex nucleus through liquid-liquid phase separation. This mechanism provides insight into the control of key cellular signature genes and offers a new perspective on the regulatory process involved in cell fate determination and disease development. Previously, it was believed that nuclear condensates formed by high concentrations of transcription factors, mediators, and RNA polymerase II through interactions of low complexity protein sequences and RNA constituted transcription hubs. Xiaowei Zhuang’s team localized hundreds of active promoters and putative enhancers [53] by multiplexed error robust fluorescent in situ hybridization (MERFISH) [125]. They proposed that multiple putative enhancer loci in the same sub-TAD with genes exhibit a spatial pattern of H3K27ac signaling similar to the spatial expression pattern of genes, i.e., indicating an enhancer hub in genomic space [125]. These putative enhancer hubs span hundreds of kilobases in genomic space and are not typically considered super enhancers. They may be associated with shadow enhancers [126] or redundant enhancers [127]. This differs somewhat from the phase separation and raises the question of whether the concept of super enhancers needs to be redefined. It remains an open question for future research whether enhancer hubs can assume a functional role similar to that of super-enhancers, such as through 3D chromatin folding.

### 4.4. Multi-Enhancer Modulation

Based on the specificity of transcriptional regulation exhibited by both TE and SE, gene regulation often involves multi-enhancers that jointly control transcription [128,129] and the enhancer hub mentioned above. We propose a model of multi-enhancer co-regulation, which plays a critical role in transcriptional regulation (Figure 3). Multi-enhancers (MEs) can be located on the same side or on different sides of the gene, and they can even be positioned far away from the gene. Different enhancers may act at different sites, resulting in various phenotypic changes. Collaborative or competitive relationships may exist among multi-enhancers, including super enhancers [56]. Therefore, understanding the molecular mechanisms of multi-enhancer regulatory patterns can provide insights into development, disease, physiology, and biology, ultimately driving advancements in precision biology.

#### 4.4.1. Multi-Enhancer Modulation Affects Biological Effects

**Figure 4:**
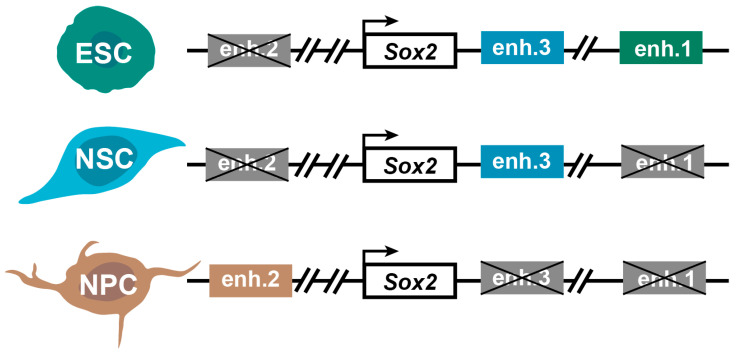
Three enhancers of the *Sox2* gene show different kinds of E-P associations. Different interaction patterns differentiate ESC into different cells. Active enhancers enh.1, enh.2, and enh.3 are shown in green, blue, and brown, respectively. Inactive enhancers are shown in gray with “×” mark. Black arrow indicates the direction of transcription initiation.

We emphasize the mediating role of enhancers in transcriptional regulation. In a multi-enhancer regulatory pattern, different enhancers can have diverse biological effects. *Sox2* is one of the three core transcription factors (*Oct4*, *Sox2*, *Nanog*) responsible for maintaining the pluripotency of embryonic stem cells (ESCs) [130,131]. These core pluripotency factors form an autoregulatory circuit and transcriptionally induce other key pluripotency genes. Understanding the transcriptional regulatory circuits of pluripotency and self-renewal in ESCs is essential to comprehending human development and harnessing the therapeutic potential of these cells due to their inherent pluripotency [132]. In mammals, distal enhancer-promoter interactions are crucial for the transcriptional activity of *Sox2*, but the mechanisms of its transcriptional regulation in ESCs have not been fully resolved, such as whether each enhancer acts individually to affect transcription or in combination to exert its function [133,134]. The question of how *Sox2* controls the direction of cell differentiation is of particular interest. Our recent study revealed different transcriptional regulation patterns of the *Sox2* gene associated with distinct enhancer-promoter interactions [11,26] (Figure 4). In a previous study, we identified three enhancers that interact with the *Sox2* promoter, each playing a unique role [11]. By utilizing the CRISPR/Cas9 system, we knocked down these three enhancers and analyzed the transcriptomes of different *Sox2* enhancer-deficient cells combined with epigenetic data such as ChIP-seq. We found that these three enhancers affect various physiological processes in stem cell differentiation, including differentiation into cardiomyocytes, early differentiation of mESCs into neuronal cells, early embryonic differentiation, and stem cell morphology maintenance. These findings suggest that different enhancers exhibit diverse transcriptional profiles [26] (Figure 4), highlighting the complex E-P association. This complex association, which synergistically regulates *Sox2* gene function in multiple ways, provides a new paradigm for the precise study of gene function and forms the basis for precision biology research.

#### 4.4.2. Complex Molecular Mechanisms of Multi-Enhancer Regulation

**Figure 5:**
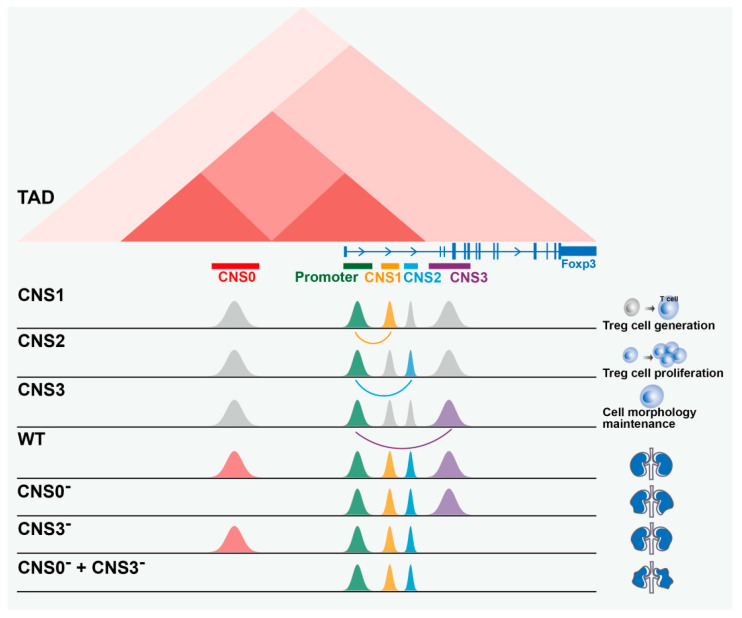
CNS0, CNS1, CNS2, and CNS3 have different effects on *Foxp3*. (1) CNS1 interacts with the *Foxp3* promoter to induce iTreg cell generation in intestine-associated lymphoid tissues. (2) *Foxp3* expression in the progeny of dividing Treg cells is dependent on CNS2, (3) CNS3 increases the frequency of Treg cells generated in the thymus and periphery and maintains cellular homeostasis. (4) Compared with wild type, deletion of CNS0 and CNS3 partially disrupted thymic Treg cell production, resulting in morphological alterations in mice. (5) Deletion of both CNS0 and CNS3 completely prevented the production of thymic Treg cells, resulting in further imbalance in tissue morphology.

Different enhancers can have distinct biological effects due to the specific molecular mechanisms involved in their regulation. The transcription factor *Foxp3*, which plays a critical role in the differentiation of regulatory T cells, is regulated by multi-enhancer elements (Figure 5). These include the conserved non-coding sequences (CNS) CNS1, CNS2, and CNS3 [135,136,137]. CNS1 contains binding sites for transcription factors such as NFAT, Smad3, and RAR/RXR and is essential for TGFβ-dependent induction of *Foxp3*. It prominently contributes to iTreg cell generation in gut-associated lymphoid tissues [138]. However, CNS1 appears to have no role in tTreg differentiation or maintenance of *Foxp3* expression [135]. On the other hand, CpG dinucleotide demethylation at CNS2 and the *Foxp3* promoter ensures stable *Foxp3* expression [139]. Acting as a classical enhancer, CNS3 influences the probability of *Foxp3* induction in precursor cells by recruiting c-Rel, a member of the NF-κB family. The exact molecular mechanism by which c-Rel binding to CNS3 initiates chromatin remodeling or contributes to the formation of a c-Rel enhancer complex at the *Foxp3* promoter is still under investigation [136]. Recent research has also revealed that *Foxp3* CNS0 and intergenic CNS3 bind unique transcription factors at early stages of thymocyte differentiation. These regions precede *Foxp3* promoter activation and are bridged by sequential genomic loops to their respective promoters. This indicates that the stratification and coordinated activation of *Foxp3* CNS0 and CNS3 initiate and stabilize *Foxp3* gene expression. This process plays a critical role in Treg cell development, maintenance, and immune self-tolerance [140]. Thus, the multi-enhancer regulation of *Foxp3* involves synergistic regulation of different enhancers, highlighting the importance of understanding the specific mechanisms involved in enhancer-promoter interactions for the precise study of gene function [138].

Earlier this year, a study investigated the macroscopic regulation of chromatin structure by a MED23 point mutation associated with intellectual disability [41]. MED23 is one of the subunits of the mediator [141]. In recently reported cases, several missense mutations of MED23 were found to be associated with familial intellectual disabilities (IDs) [142,143,144]. The study found that the MED23 c.G1850A (p.R617Q) point mutation leads to abnormal expression of a series of genes related to learning and memory. The molecular mechanism was identified as the MED23 R617Q point mutation downregulating gene expression by specifically reducing enhancer activity. This reduction weakens enhancer-promoter interactions and alters three-dimensional chromatin conformation. Consequently, the expression of the *DACH1* (a cell fate determination factor [145]) is downregulated, ultimately resulting in the abnormal elevation of immediate-early genes (IEGs) *FOS* and *JUN* expression. These IEGs are closely related to learning and memory and can trigger mental retardation. Interestingly, regulatory elements both upstream and downstream of this gene showed changes in activity, although the authors did not specifically explain the molecular mechanisms involved. This study demonstrates the role of mediator in the topology of enhancers in transcriptional regulation.

Our limited understanding of the molecular mechanisms of transcriptional regulation suggests that refining genome-wide annotation remains a priority a comprehensive understanding of chromatin structure and transcriptional regulation. Another study earlier this year also pointed out the synergistic and mutually exclusive relationships between super-enhancers of the same locus of action, which has prompted our thinking. Therefore, based on the complexity of molecular composition, we question the existing concept of super enhancers. Is it possible that super enhancers actually belong to a multi-enhancer regulatory model but are simply classified as a cluster of enhancers due to our lack of knowledge and annotation? All of this remains to be discovered. However, we believe that with the development of precision biology, the aforementioned questions will hopefully be gradually solved.

## 5. Summary and Outlook

In the field of precision biology, the multi-enhancer regulatory model has progressively demonstrated its importance. The complex biological processes are expected to be explained by the resolution of enhancers. Despite the advancements in 3D genomics technology, single-cell technology, and imaging technology, accurately describing the molecular mechanism of enhancer-mediated transcriptional regulation remains challenging [10,146]. Although recent technologies like STARR-seq [45,147], chromatin accessibility-based DNase-seq [148], nicking enzyme assisted sequencing (NicE-seq) [149] allow for the identification of more enhancer sequences, predicting and identifying enhancers still pose difficulties (Figure 1). This is primarily because enhancer sequences are not as highly conserved as promoter sequences, and enhancers are usually located in non-coding regions near genes [11]. Even though it is generally believed that the function of enhancers is not related to their precise location, distance, or orientation, studies have shown that ultra-conserved enhancers are often enriched nearest target genes (tens to hundreds of kilobases) among species [150,151]. Nevertheless, the functional role of enhancers is broadly conserved across animal species because of the flexible “regulatory grammar” of enhancers [152]. Theoretically, even if the number, arrangement, or type of motifs that constitute enhancers change, they can maintain functional conservation across species [152], which provides a foundation for exploring a generalized model of enhancer regulation. Additionally, an enhancer may interact with multiple target genes, and determining the true targets of an enhancer presents a challenge. Furthermore, different enhancers may collaborate to regulate a gene [26,56], and the regulatory mechanism underlying this collaboration is not fully understood. The multi-enhancer regulatory model we propose may provide a framework to explain this regulatory mechanism, and we hope that future researchers will conduct more in-depth studies in this direction.

The interaction between enhancers and target genes is predominantly achieved through the three-dimensional structure of the genome [146]. However, our understanding of the three-dimensional genome structure is still insufficient, and further research is needed to gain a better understanding of the formation and dynamic changes of the three-dimensional genome structure, as well as the interaction between enhancers and target genes. In addition, as the technology continues to mature and data volumes increase, addressing important questions such as ensuring data quality, accurately extracting key information from large datasets, and selecting appropriate visualization tools for three-dimensional genomics (e.g., heat maps like Juicebox, epigenome browsers, and disease mutation associations like 3Disease Browser) are essential [153,154]. Furthermore, while single-cell imaging technology now enables us to observe the dynamic formation of DNA loops and long-distance interactions of regulatory elements within an individual cell, research on enhancers in precision biology is still limited to cell lines or mouse models, which may not fully capture the complexity of tissues or organs [37]. In summary, there is an urgent need to develop new technologies and methods to further investigate the role of enhancers in transcriptional regulation and to support the analysis of gene function at the whole-genome level. Enhancer-mediated transcriptional regulation represents a highly complex and challenging area of research, and the advancement of precision biology requires scientists to continually develop new technologies and methods to address the existing challenges.

Although we have only summarized the research in transcriptional regulation, especially the involvement of enhancers, in precision biology and three-dimensional genomics here, the technical and analytical methods of precision biology undoubtedly have the potential to unravel the mechanisms of gene regulation, signal transduction, metabolic pathways, and other complex physiological processes at the molecular level. These advancements can greatly contribute to various fields, such as basic biology, medicine, and animal and plant breeding. For example, the comprehensive and accurate analysis of patients using high-throughput techniques and bioinformatics methods can provide value to assist doctors in developing personalized diagnoses and treatment plans [155,156]. Gaining a precise and in-depth understanding of the interaction between drugs and organisms will accelerate the development of new drugs while reducing research and development costs and risks [1,2,157]. Notably, there are ongoing experiments utilizing AI and ML for COVID-19 drug repurposing, which addresses the urgent needs presented by the COVID-19 pandemic [31]. By analyzing individual genomic, transcriptomic, proteomic, and other information, it becomes possible to predict disease incidence [27]. The advantages of precision biology in revealing the fundamental mechanisms of life also hold the potential groundbreaking advancements in Adoptive T-cell therapy (ACT) based on synthetic biology theories. This offers new perspectives on modifying T cells using tumor-specific T cell receptors (TCRs) or chimeric antigen receptors (CARs) [158,159,160]. Incorporating 3D genomics information into cell fate decisions, it becomes possible to reprogram terminally differentiated somatic cells into induced pluripotent stem cells (iPS cells) by introducing specific transcription factors [161,162,163]. Combining targeted cell editing with enhancer-mediated transcriptional regulatory mechanisms may enable the direct transformation of cancer cells into normal cells in the future. The development of precision biology will also bring new opportunities and technologies to animal and plant breeding. For example, using CRISPR/Cas9 technology to edit specific genes in animals and plants can modify traits such as growth rate, disease resistance, and yield, thereby optimizing breeding outcomes [7]. Moreover, when combined with bioinformatics technology, it becomes possible to predict and simulate gene expression patterns and regulatory mechanisms in animals and plants, providing vital theoretical support for breeding and improving the efficiency and quality of agricultural production. Noteworthy studies have been conducted to analyze the genetic correlation of several economic traits in pigs based on Genome-wide association study (GWAS) data [164]. Such studies offer a theoretical foundation for genetic control of fatty acid composition in pigs, co-regulation of other economic traits, and the design of breeding strategies for manipulating fatty acid composition in pigs. In addition to applications in domestic animals such as pigs [164] and cattle [165], GWAS is also used in the breeding of pets such as cats [166]. Moreover, in crops such as rice, barley, and wheat, the design of high-density and high-quality SNP (Single Nucleotide Polymorphisms) arrays using GWAS is becoming increasingly feasible [167,168], and it is anticipated play a crucial role in plant functional genomics studies and molecular breeding.

Finally, we hope to explain the concept and principles of precision biology beyond the scope of transcriptional regulation. The development of precision biology requires scientists to improve various foundational theories, such as gene function at the whole genome level, different patterns of gene co-regulation, chromatin conformation, and intracellular biological processes. Additionally, we are pleased to acknowledge that the advancements in three-dimensional genomics can provide a powerful link for studying the dynamic nature of diverse biological processes. Furthermore, integration and collaboration across multiple disciplines, including imaging, genomics, computational science, and physics, are essential. Precision biology instills great optimism in advancing our comprehension of gene regulation and cellular processes. The development of precision biology, grounded in molecular mechanisms, will inevitably drive progress across a wide range of scientific disciplines and industries. Ultimately, this will contribute to a deeper understanding of the natural world and have a positive impact on individuals and society as a whole.

## Data Availability

No new data were created.
